# Measurement of airway function using invasive and non-invasive methods in mild and severe models for allergic airway inflammation in mice

**DOI:** 10.3389/fphar.2014.00190

**Published:** 2014-08-12

**Authors:** Kim A. T. Verheijden, Paul A. J. Henricks, Frank A. Redegeld, Johan Garssen, Gert Folkerts

**Affiliations:** ^1^Division of Pharmacology, Faculty of Science, Utrecht Institute for Pharmaceutical Sciences, Utrecht UniversityUtrecht, Netherlands; ^2^Immunology, Nutricia ResearchUtrecht, Netherlands

**Keywords:** airway hyperresponsiveness, airway inflammation, lung resistance, Penh

## Abstract

In this study a direct comparison was made between non-invasive and non-ventilated unrestrained whole body plethysmography (Penh) (conscious animals) and the invasive ventilated lung resistance (R_L_) method (anesthetized animals) in both mild and severe allergic airway inflammation models. Mild inflammation was induced by intraperitoneal sensitization and aerosols of ovalbumin. Severe inflammation was induced by intraperitoneal sensitization using trinitrophenyl-ovalbumin, followed by intranasal challenges with IgE-allergen complexes. A significant increase in airway responsiveness to methacholine was observed in the mild inflammation group when R_L_ was measured. Significant changes in both R_L_ and Penh were observed in the severe inflammation groups. There was a significant increase in the number of inflammatory cells in the Broncho-Alveolar Lavage Fluid (BALF) in both the mild and severe inflammation animals. The enforced ventilation of the animals during the R_L_ measurement further increased the number of cells in the BALF. IL-2 and RANTES levels in the BALF were higher in the severe inflammation groups compared to the mild inflammation groups. Penh gave only reliable measurements during severe airway inflammation. Measuring R_L_ gave consistent results in both mild and severe allergic airway inflammation models however, ventilation induced an additional cell influx into the airways.

## Introduction

Asthma is characterized by airway hyperresponsiveness and inflammation. Airway inflammation is initiated and propagated by multiple inflammatory mediators such as lipid mediators, cytokines, and chemokines (O'Byrne and Inman, 2003; Barnes, 2008). To investigate airway function in preclinical models both non-invasive and invasive analysis methods have been used. Unrestrained whole body plethysmography (Penh), a non-invasive method for measurement of airway responsiveness, has been used frequently but its validity is under debate. For this method the airway function is measured with enhanced pause (Penh), an empirical and dimensionless parameter (Frazer et al., 2011). Increased bronchoconstriction is considered to be paralleled with an increase in Penh. However, various experimental conditions resulting in a change of breathing pattern can also affect Penh (Sly et al., 2004). Penh is viewed as a better indicator for control of breathing (as seen in respiratory patterns) rather than an indicator for mechanical lung function (Bates and Irvin, 2003; Irvin and Bates, 2003; O'Byrne and Inman, 2003; Adler et al., 2004). Respiratory patterns can be influenced by stress Hoymann (2007), heating or humidification of the chamber, affecting the measured signal (Lundblad et al., 2002; Adler et al., 2004). Moreover, the outcome of unrestrained Penh may be dependent on the mouse strain used (Adler et al., 2004). Furthermore, unrestrained Penh measures changes in the upper airway parts (nose) as well as in the lower airways - of particular importance in rodents as these animals are nose-breathers (Hoymann, 2006). There are also practical advantages of Penh measurements because the animals do not need to be anesthetized and do not need surgery for ventilation hence, the method is simpler than others and less time-consuming (Bates and Irvin, 2003; Irvin and Bates, 2003; Berndt et al., 2011; Hoymann, 2012). Also, the animal can be used for repeated measurements in time (Albertine et al., 2002; Irvin and Bates, 2003; Adler et al., 2004; Lomask, 2006; Berndt et al., 2011). Invasive lung resistance measurement (R_L_) is another method for measuring airway function. Although R_L_ measurements are considered to more accurately represent lung function, the method also has its limitations. First, the animals have to be anesthetized which might change physiological parameters such as body temperature. A cannula also has to be placed into the trachea which could cause local mechanical stress. Moreover, the animals are artificially ventilated with a fixed volume which artificially influences the pattern of breathing and might have an effect on the homeostasis of the airways. The technique is also time-consuming (Glaab et al., 2007) and terminal for the animals after measurement. There is however, no stress for the animal during the experiment (Hoymann, 2006, 2012), and exposing the lower airways to allergens or agonists is more accurate via a cannula in the trachea. When the trachea is orally intubated, instead of with an incision, repetitive measurements can be conducted in spontaneously breathing mice (Brown et al., 1999; Glaab et al., 2004; De Vleeschauwer et al., 2011). The most important advantage of the resistance method is that it is a sensitive and specific measurement to analyze pulmonary mechanics (Glaab et al., 2007; Hoymann, 2012). Due to the continuous discussion on the measurement of lung function in rodents, a comparative study between the non-invasive measurement airway function (Penh) and the invasive measurement airway function [Resistance (R_L_)] was conducted in mild and severe allergic airway inflammation models, which mimics some features of allergic asthma in humans.

## Materials and methods

### Mice

Male BALB/c mice (Charles River, Maastricht, The Netherlands), 6–8 weeks old (20–25 g), were used in all experiments. Mice were maintained under standard laboratory conditions. Food and water were provided *ad libitum*. All animal experiments were conducted in compliance with the Guidelines of the Ethical Committee on the Use of Laboratory Animals of the University Utrecht.

### Sensitization and airway challenge

#### Mild airway inflammation model

**Figure 1:**
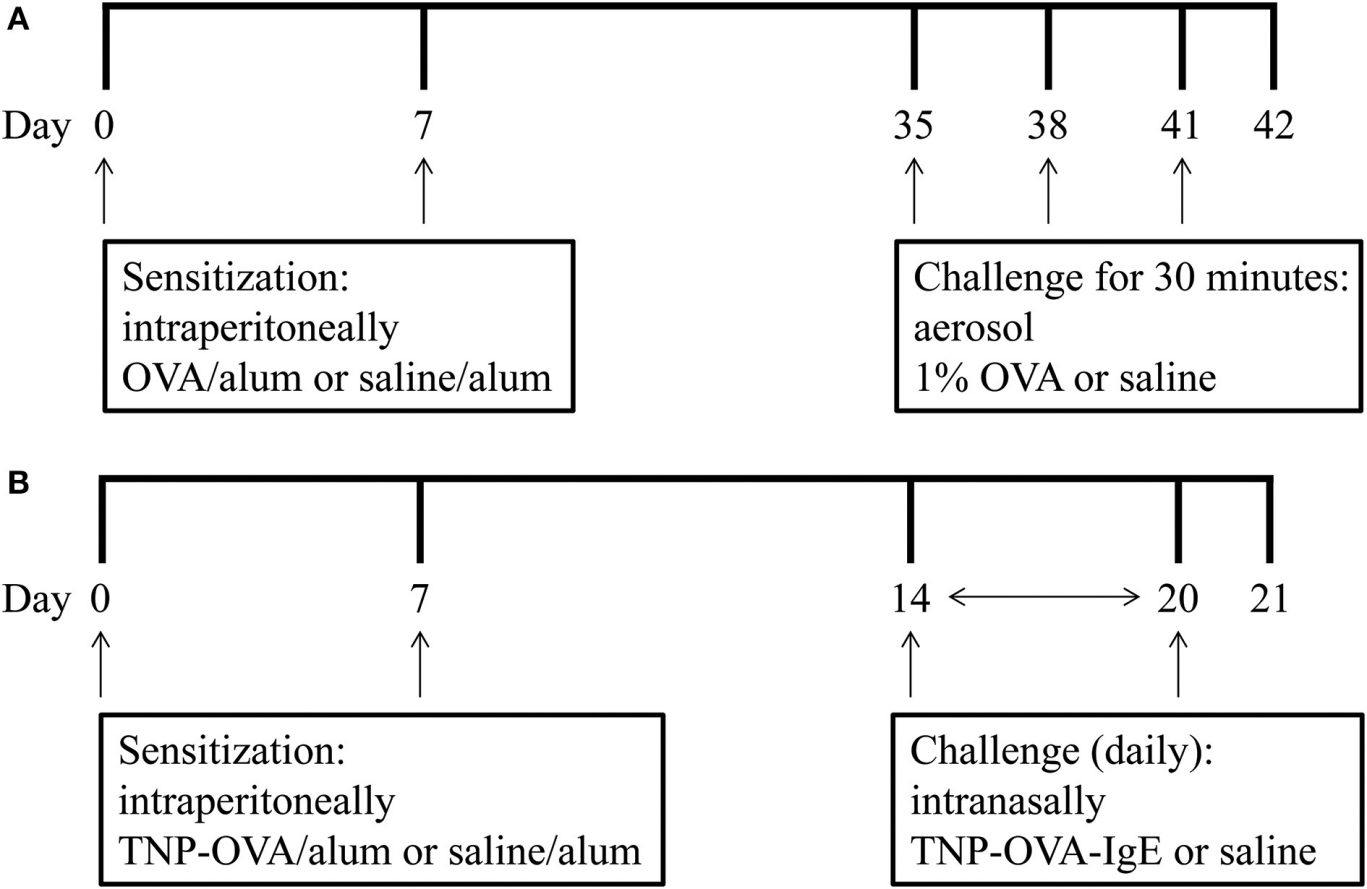
**Experimental scheme of the mild airway inflammation model (A) and severe airway inflammation model (B)**.

On days 0 and 7 mice were sensitized with ovalbumin (OVA; chicken egg albumin, grade V, Sigma, St. Louis, MO, USA) or treated with saline. Active sensitization was conducted by two intraperitoneal injections of 0.1 mL alum-precipitated antigen, comprising 10 μ g OVA absorbed into 2.25 mg alum (AlumImject; Pierce, Rockford, IL, USA). On days 35, 38, and 41 mice were exposed either to an OVA (1% ovalbumin in pyrogen-free saline, OVA group) or control solution (saline, SAL group) aerosol challenge for 30 min. The aerosol was conducted in a plexiglass exposure chamber (5 L) coupled to a Pari LC Star nebulizer (PARI Respiratory Equipment, Richmond, VA, USA; particle size 2.5–3.1 μm) driven by compressed air at a flow rate of 6 L/min (Ten Broeke et al., 2006) (Figure 1A). An overview of the groups included in this study is given in Table 1.

**Table 1:** **Mouse groups included in the mild airway inflammation model**.

**Sensitization**	**Challenge**	**Group abbreviation**
SAL	SAL	SAL-SAL
SAL	OVA	SAL-OVA
OVA	SAL	OVA-SAL
OVA	OVA	OVA-OVA

SAL, saline; OVA, ovalbumin.

#### Severe airway inflammation model

On days 0 and 7 mice were sensitized with trinitrophenyl (TNP) conjugated-ovalbumin (OVA; chicken egg albumine, grade V, Sigma, St. Louis, MO, USA) or saline. Sensitization was conducted by two intraperitoneal injections of 0.1 mL alum-precipitated antigen, comprising 10 μ g TNP-OVA absorbed into 2.25 mg alum (AlumImject; Pierce, Rockford, IL, USA) or saline. On days 14–20, mice were challenged daily by intranasal administration of a TNP-ovalbumin/IgE immune complex [2 μ g TNP-OVA plus 20 μ g DNP-specific IgE (clone H1 26.82)] or saline, as described by Pasquier et al. (2005) and Sagar et al. (2013) (Figure 1B). An overview of the groups included in this study is given in Table 2.

**Table 2:** **Mouse groups included in the severe airway inflammation model**.

**Sensitization**	**Challenge**	**Group abbreviation**
SAL	SAL	SAL-SAL
SAL	TNP-OVA-IgE	SAL–TNP-OVA-IgE
TNP-OVA	SAL	TNP-OVA–SAL
TNP-OVA	TNP-OVA-IgE	TNP-OVA–TNP-OVA-IgE

SAL, saline; TNP-OVA, trinitrophenyl conjugated-ovalbumin; TNP-OVA-IgE, trinitrophenyl conjugated-ovalbumin /IgE immune complex.

### Measurement of airway responsiveness *in vivo*

#### Non-invasive measurement airway function (Penh)

Airway responsiveness was measured 24 h after the last aerosol exposure by recording respiratory pressure curves using barometric unrestrained whole-body plethysmography (Buxco; EMKA Technologies, Paris, France) in response to inhaled methacholine (acetyl-β-methyl-choline chloride, Sigma, The Netherlands) in conscious unrestrained mice. Airway responsiveness was expressed as enhanced pause (Penh) as described in detail previously (Hamelmann et al., 1997). Briefly, mice were placed in a whole-body chamber and basal readings were obtained and averaged for a 3 min period. Subsequently, increasing doses of methacholine (0–50 mg/mL), were aerosolized for 3 min, and readings were taken and averaged for 3 min after each nebulization (Vos et al., 2007).

#### Invasive measurement airway function (resistance (R_L_))

The mice were intraperitoneally anesthetized with KM-mix (containing Ketamine (Vetoquinol S.A., France; 125 mg/kg) and Medetomidine (Pfizer, Netherlands; 0.4 mg/kg).The animals were ventilated [O_2_/air (1:2)] at a frequency of 150 beats/min (*TV* = 0.3 mL). An anesthesia-induced fall in body temperature was avoided by placing the animals in a heated box in which the body temperature was kept at 37°C. The mice were prepared for the measurement of lung parameters [pulmonary resistance (R_L_)]. Pressure was determined as follows: a small catheter was placed in the trachea of the mouse. This catheter was connected to a pressure transducer fixed on the box (EMKA Technologies, Paris, France) and transpulmonary pressure was determined by measuring pressure differences in the cannula in the trachea. Airflow and tidal volume were determined using a flow transducer fixed to the body box that measured flow differences inside the box. Increasing doses of methacholine (acetyl-β-methyl-choline chloride, Sigma) (0–50 mg/mL, 10% puff for 10 s) were administered by aerosol generated in a nebulizer (EMKA Technologies, Paris, France) connected in between the animal in the body box and the ventilator (EMKA Technologies, Paris, France). After the first dose of methacholine, pulmonary resistance (R_L_) was measured for 3 min and this procedure was repeated for all doses. R_L_ was yielded by dividing transpulmonary pressure by airflow at isovolume points. Data are presented as average R_L_ in cm H_2_O/mL^*^s^−1^ (Sagar et al., 2014a).

### Bronchoalveolar lavage

Mice were killed by an intraperitoneal overdose of pentobarbital (Nembutal™, Ceva Santé Animale, Naaldwijk, The Netherlands) after the airway responsiveness measurement. The trachea was trimmed free of connective tissue and a small incision was made for insertion of a cannula into the trachea. Lungs were lavaged with 1 mL of pyrogen-free saline (0.9% NaCl, 37°C) supplemented with protease inhibitor cocktail tablet. The supernatant of the first mL was used for cytokine and chemokine measurement. Afterwards the lungs were lavaged 3 times with 1 mL saline solution (0.9% NaCl, 37°C). The BAL cells were centrifuged (400 g, 5 min) and pellets of the four lavages were pooled, resuspended, and total numbers of BAL cells were counted by use of a Bürker-Türk chamber. For differential BAL cell counts cytospin preparations were made and stained with Diff-Quick (Merz and Dade A.G., Düdingen, Switzerland). After coding, all cytospin preparations were evaluated by one observer using oil immersion microscopy. Cells were differentiated into macrophages, lymphocytes, neutrophils, and eosinophils by standard morphology. At least 200 cells per cytospin preparation were counted and the absolute number of each cell type was calculated (Braber et al., 2011).

### Measurement of cytokines and chemokines

A standard mouse cytokine 21-plex assay (GM-CSF, IFNγ, IL-10, IL-12p40, IL-12p70, IL-13, IL-17, IL-1β, IL-2, IL-4, IL-5, IL-6, IL-9, IP-10, KC, MCP-1, MIG, MIP-1α, MIP-2, RANTES, and TNFα, Luminex; Biosource, Invitrogen, Breda, The Netherlands) was used to determine cytokine and chemokine concentrations in the BALF (*n* = 4–5) according to the manufacturer's instructions (Braber et al., 2010). The concentrations of these cytokines and chemokines were expressed as pg/mL.

### Lung histology

After lung lavage, lungs were fixated with 10% formalin infusion through the cannula at a constant pressure of 25 cm H_2_O. After at least 24 h of fixation lungs were embedded in paraffin. After embedding, 5 μ sections were cut and stained with hematoxylin/eosin (H and E) according to standard methods. Photomicrographs were taken with an Olympus B × 50 microscope equipped with a Leica DFC 320 digital Camera (Akbari et al., 2014; Sagar et al., 2014b).

### Statistical analysis

Results are presented as the mean ± standard error of mean (SEM). Data were statistically analyzed using a One-Way ANOVA followed by a Bonferroni *post-hoc* analysis. *P*-values < 0.05 were considered to be significant. Statistical analyses were conducted using GraphPad Prism software (version 5.0).

## Results

### Airway function

Airway responsiveness was measured in conscious unrestrained mice (Penh) or in anesthetized ventilated animals (R_L_) exposed to inhaled methacholine 24 h after the last OVA or saline challenge.

#### Measurement of airway function in the mild airway inflammation model

**Figure 2:**
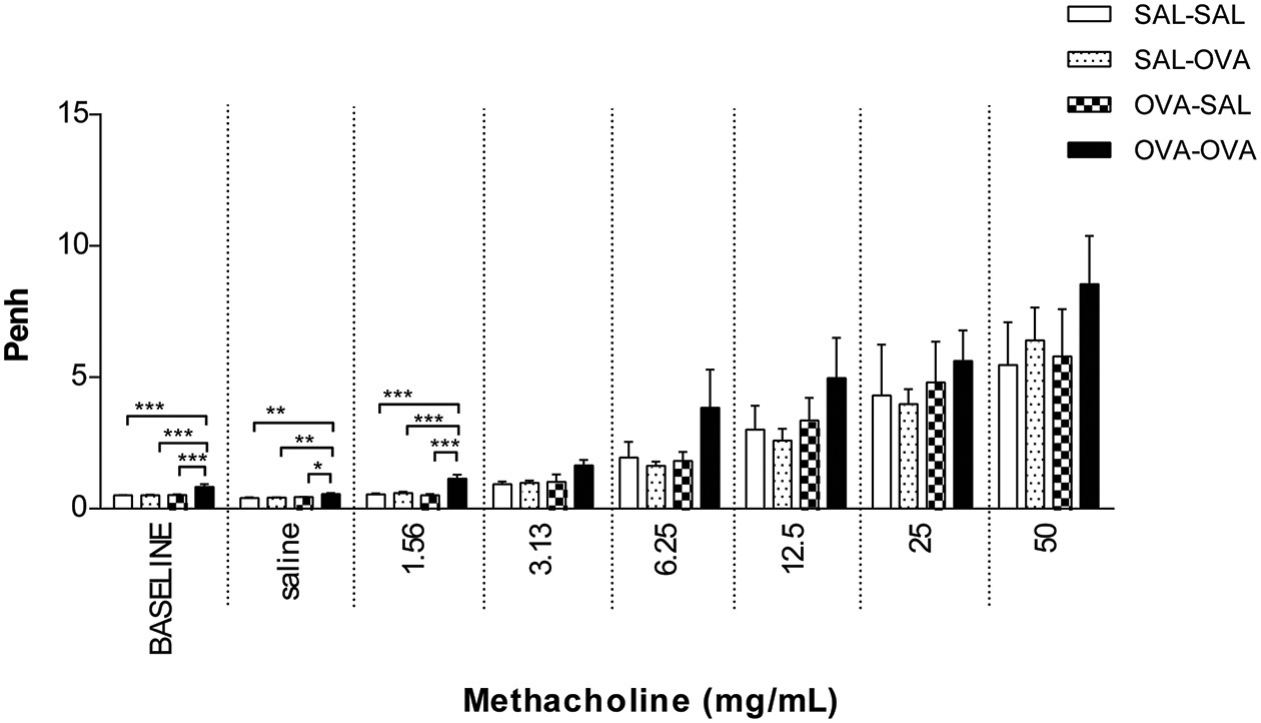
**Airway responsiveness (Penh) to methacholine in the mild airway inflammation model**. Airway responsiveness was measured in mice sensitized with saline or ovalbumin and challenged by aerosol with saline or ovalbumin. Values are expressed as mean ± s.e.m. ^*^P < 0.05, ^**^P < 0.01, ^***^P < 0.001; using a One-Way ANOVA followed by a Bonferroni *post-hoc* analysis, *n* = 5–9 mice/group.

**Figure 3:**
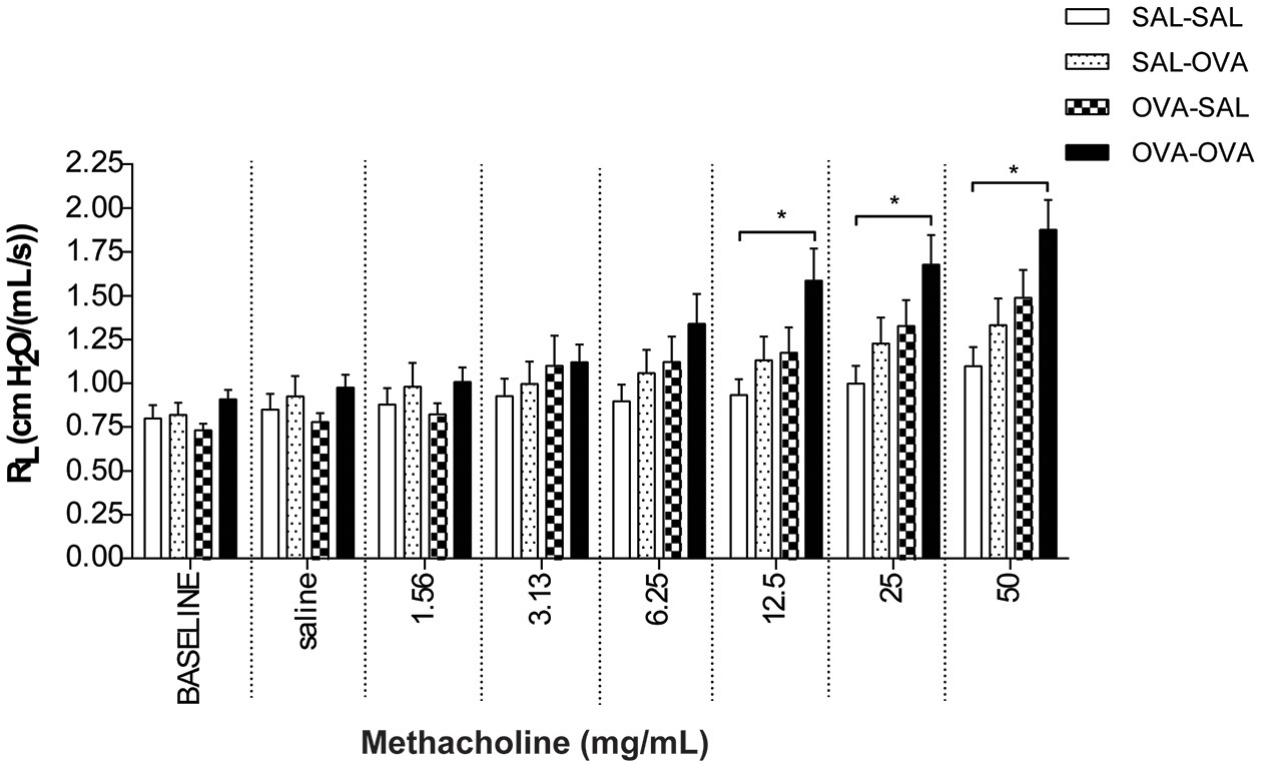
**Resistance measurement (R_L_) to methacholine in the mild airway inflammation model**. Resistance was measured in ventilated mice sensitized with saline or ovalbumin and challenged by aerosol with saline or ovalbumin. Values are expressed as mean ± s.e.m. ^*^P < 0.05; using a One-Way ANOVA followed by a Bonferroni *post-hoc* analysis, *n* = 7–9 mice/group.

In unrestrained mice, basal airway resistance was significantly increased in the OVA-OVA group compared to the SAL-SAL group (Figure 2). Moreover, there was an increase in Penh observed after administrating a low dose of methacholine to the OVA-OVA group compared to the SAL-SAL group. However, this effect was not observed using higher doses of methacholine. The Penh dose-dependently increased in the SAL-SAL group in response to methacholine inhalation to a maximum of 5.47 ± 1.63. The Penh in the OVA-OVA group was increased by 56% to a maximum of 8.54 ± 1.84, but this increase was not significant compared to the SAL-SAL group (Figure 2). In ventilated mice, basal airway resistance was not significantly different between the experimental groups (Figure 3). Methacholine slightly increased the R_L_ in the SAL-SAL group, while the R_L_ was significantly increased by 71% in the OVA-OVA group after methacholine (12.5–50 mg/mL) inhalation (Figure 3).

#### Measurement of airway function in the severe airway inflammation model

**Figure 4:**
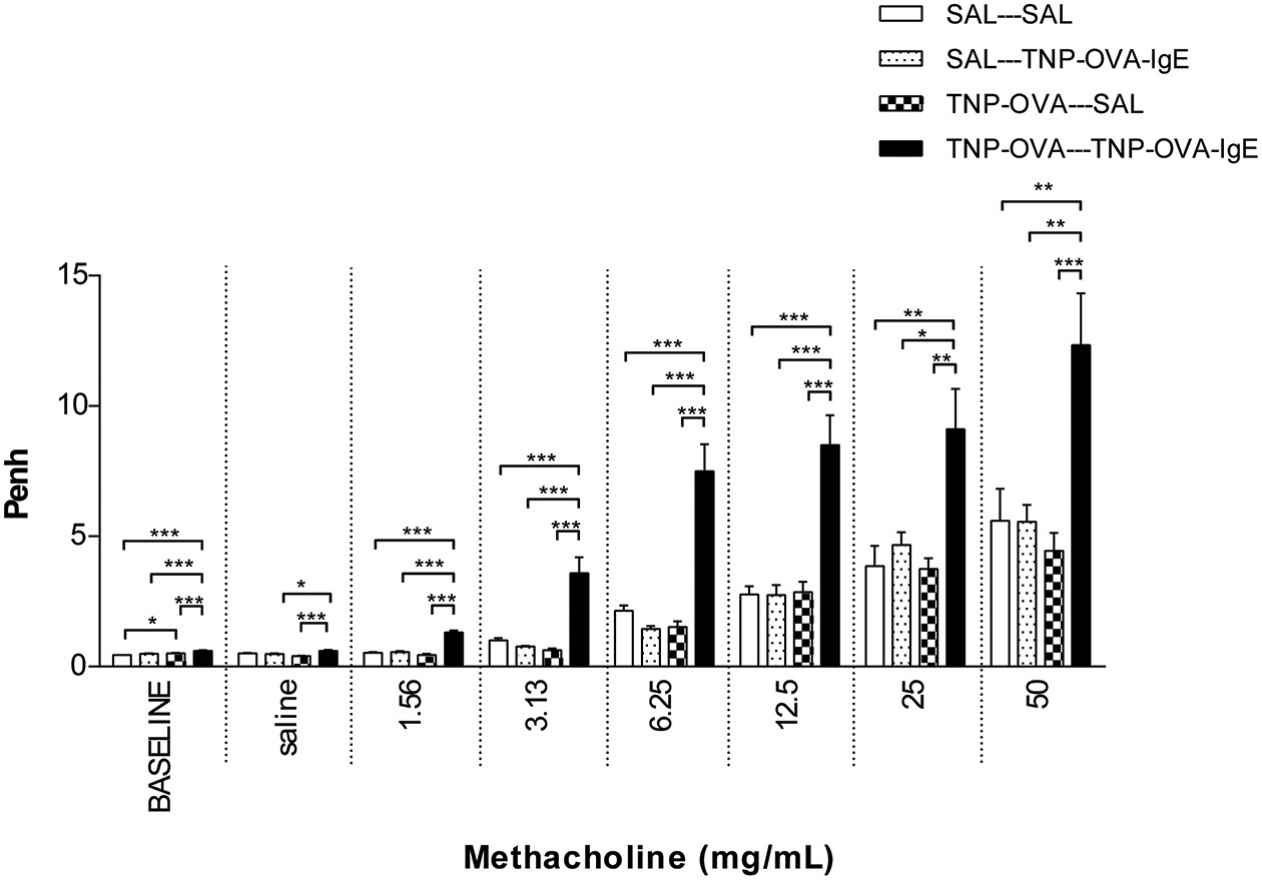
**Airway responsiveness (Penh) to methacholine in the severe airway inflammation model**. Airway responsiveness was measured in mice sensitized with saline or TNP-OVA and challenged intranasally with saline or TNP-OVA-IgE. Values are expressed as mean ± s.e.m. ^*^P < 0.05, ^**^P < 0.01, ^***^P < 0.001; using a One-Way ANOVA followed by a Bonferroni *post-hoc* analysis, *n* = 7–9 mice/group.

**Figure 5:**
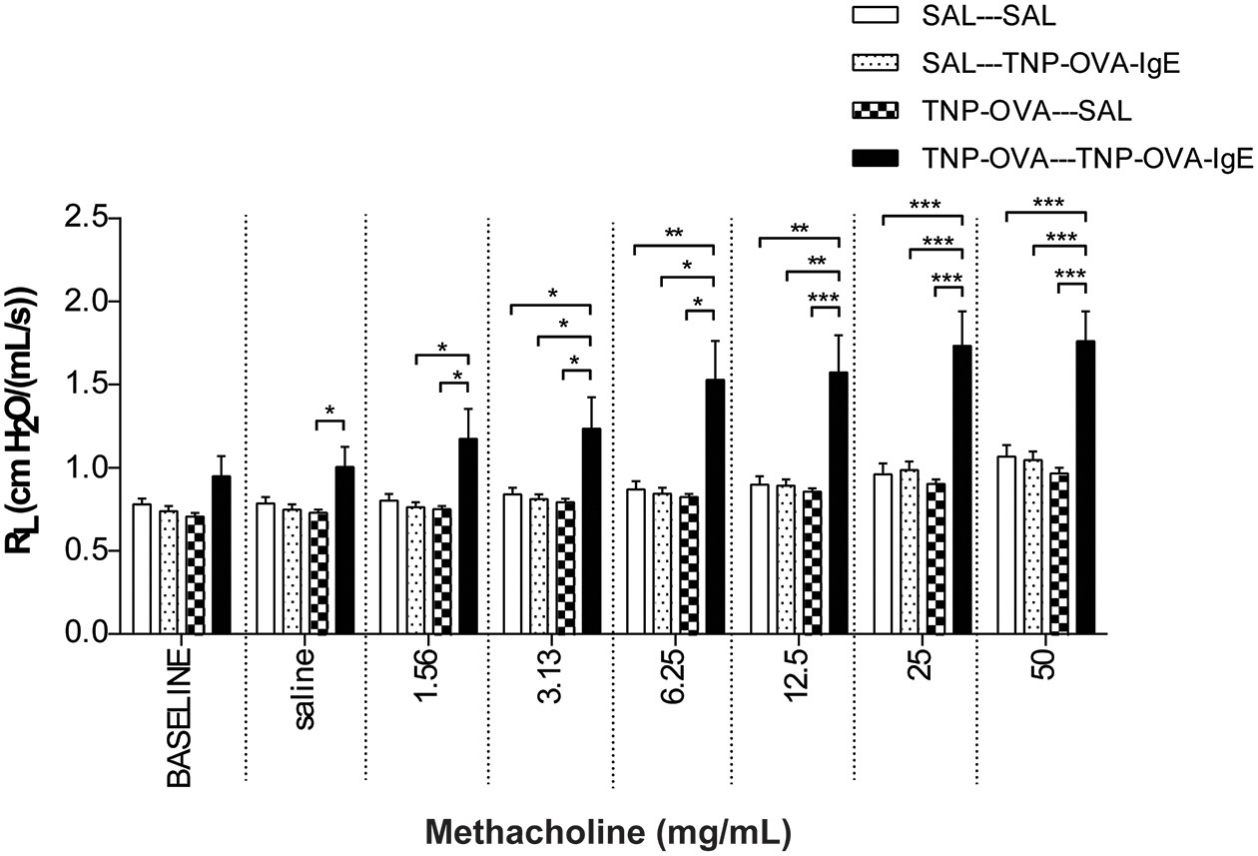
**Resistance measurement (R_L_)to methacholine in the severe airway inflammation model**. Resistance was measured in ventilated mice sensitized with saline or TNP-OVA and challenged intranasally with saline or TNP-OVA-IgE. Values are expressed as mean ± s.e.m. ^*^P < 0.05, ^**^P < 0.01, ^***^P < 0.001; using a One-Way ANOVA followed by a Bonferroni *post-hoc* analysis, *n* = 9 mice/group.

Basal airway resistance was significantly increased in the TNP-OVA—TNP-OVA-IgE group compared to the SAL-SAL group. The Penh increased dose-dependently in the SAL-SAL group after methacholine inhalation (Figure 4). The Penh in the TNP-OVA—TNP-OVA-IgE group was significantly increased compared to the SAL-SAL group to a maximum of 12.33 ± 1.99 (Figure 4). The increase in Penh was 1.5 times higher compared to the sensitized and challenged animals with mild airway inflammation (Figure 2). Although the basal R_L_ did not differ in the OVA-OVA group of the mild model (Figure 3), basal R_L_ tended to increase in the severe model (Figure 5), reaching the level of significance after saline exposure. As in the mild model, methacholine slightly increased the R_L_ in the SAL-SAL group of the severe model. The R_L_ was significantly increased in the TNP-OVA—TNP-OVA-IgE group after the methacholine inhalation to a maximum of 1.76 ± 0.18 cm H_2_O/mL^*^s^−1^. The maximal increase in R_L_ to methacholine in the severe model was a level similar to the OVA-OVA group in the mild airway inflammation model, but the sensitivity was higher as evidenced by significant changes at lower doses of methacholine (Figures 3, 5).

### Airway inflammation in the mild model

The influx of inflammatory cells (total and differentiated cell numbers) in the lungs was measured after lung function measurement. Total BAL cell numbers were 4 times higher in the OVA-OVA group compared to the SAL-SAL group after the Penh measurement (Table 3). This increase in total cells was mainly due to an increase in the number of eosinophils. Moreover, the number of lymphocytes in the OVA-OVA group was significantly increased. The total number of inflammatory cells after R_L_ measurement was nine times higher in the OVA-OVA group compared to the SAL-SAL group. Lymphocytes, neutrophils, and eosinophils were all significantly increased in the OVA-OVA group. There were no differences between the SAL-SAL groups after measuring Penh or R_L_. In contrast, after R_L_ measurement, the total number of BAL cells in the OVA-OVA group was three times higher compared to the OVA-OVA group after Penh measurement. This increase was mainly due to a significant increase in the number of eosinophils and to a lesser extent to an increase of lymphocytes and neutrophils (Table 3).

**Table 3:** **Total and differential cell counts in broncho-alveolar lavage fluid in the mild airway inflammation model**.

	Bronchoalveolar cells (*10^4^/ml) (mean ± s.e.m.)					
	**Groups**	**Total**	**Macrophages**	**Lymphocytes**	**Neutrophils**	**Eosinophils**
Penh	SAL-SAL	23.9 ± 1.0	23.6 ± 1.0	0.1 ± 0.1	0.05 ± 0.03	0
	SAL-OVA	25.2 ± 2.4	25.0 ± 2.4	0.02 ± 0.01	0.2 ± 0.1	0
	OVA-SAL	26.5 ± 3.5	26.3 ± 3.5	0.08 ± 0.04	0.1 ± 0.1	0
	OVA-OVA	95.2 ± 30.6	35.4 ± 5.4	4.9 ± 2.0	9.1 ± 5.2	45.7 ± 20.1^*^
R_L_	SAL-SAL	31.9 ± 2.6	31.5 ± 2.5	0.07 ± 0.04	0.4 ± 0.1	0
	SAL-OVA	33.8 ± 3.8	33.5 ± 3.8	0.06 ± 0.04	0.3 ± 0.1	0
	OVA-SAL	31.9 ± 3.3	30.2 ± 2.9	0.2 ± 0.06	1.6 ± 0.28	0.4 ± 0.2
	OVA-OVA	291.8 ± 47.1^***^^###^	50.1 ± 6.7	22.6 ± 4.7^***^^###^	20.1 ± 2.9^***^^#^	199.0 ± 37.5^***^^###^

Values are expressed as mean ± s.e.m.

* *P < 0.05*,

*** *P < 0.001; significantly different from the saline-saline group*,

# *P < 0.05*,

### *P < 0.001 significant different from the OVA-OVA Penh group, using a One-Way ANOVA followed by a Bonferroni post-hoc analysis*.

### Airway inflammation in the severe model

The total number of BAL cells after the Penh measurement in the SAL-SAL group of the severe model did not differ from the number of cells after the R_L_ measurement (Table 4) and showed a slight increase compared to Penh and R_L_ measurement in the mild model (Table 3). Daily intranasal administration of TNP-OVA-IgE from day 14–20 of the SAL group significantly increased the number of BAL cells whereas intranasal saline administration had no effect. This increase was mainly due to an increase in macrophages and neutrophils and not caused by changes in the number of eosinophils (Table 4). The total number of BAL cells was increased in the TNP-OVA—TNP-OVA-IgE group compared to the SAL-SAL group after the Penh measurement (Table 4). The total number of BAL cells was five times higher than in the mild OVA-OVA group (Table 3). This increase was mainly due to an increase in macrophages and eosinophils. The total number of BAL cells consisted for nearly 65% of eosinophils. Again the number of inflammatory cells was the highest in the severe group after ventilation of the animals (Table 4).

**Table 4:** **Total and differential cell counts in broncho-alveolar lavage fluid in the severe airway inflammation model**.

	Bronchoalveolar cells (*10^4^/ml) (mean ± s.e.m.)					
	**Groups**	**Total**	**Macrophages**	**Lymphocytes**	**Neutrophils**	**Eosinophils**
Penh	SAL-SAL	43.1 ± 8.1	42.9 ± 8.1	0.03 ± 0.03	0	0
	SAL—TNP-OVA-IgE	156.8 ± 23.3^***^	125.1 ± 16.8^***^	1.89 ± 0.8	29.8 ± 8.4^**^	0
	TNP-OVA—SAL	42.7 ± 4.4	42.1 ± 4.4	0.27 ± 0.2	0.25 ± 0.1	0
	TNP-OVA—TNP-OVA-IgE	552.8 ± 24.7^***^	185.7 ± 9.7^***^	9.63 ± 2.7^***^	2.66 ± 1.6	354.8 ± 26.2^***^
R_L_	SAL-SAL	43.1 ± 6.3	42.8 ± 6.3	0.09 ± 0.1	0.23 ± 0.1	0
	SAL—TNP-OVA-IgE	209.8 ± 31.6^**^	148.75 ± 21.3^**^	4.6 ± 1.8	56.5 ± 14.4^***^	0
	TNP-OVA—SAL	42.2 ± 5.6	41.8 ± 5.6	0.2 ± 0.1	0.1 ± 0.1	0
	TNP-OVA—TNP-OVA-IgE	667.5 ± 56.9^***^	211.9 ± 37.5^***^	26.5 ± 6.2^***^^###^	9.2 ± 5.5	419.9 ± 35^***^^#^

Values are expressed as mean ± s.e.m.

** *P < 0.01*,

*** *P < 0.001; significantly different from the saline-saline group*,

# P < 0.05;

### P < 0.001 significant different from the TNP-OVA—TNP-OVA-IgE Penh group, using a One-Way ANOVA followed by a Bonferroni post-hoc analysis.

### Cytokine measurements

**Figure 6:**
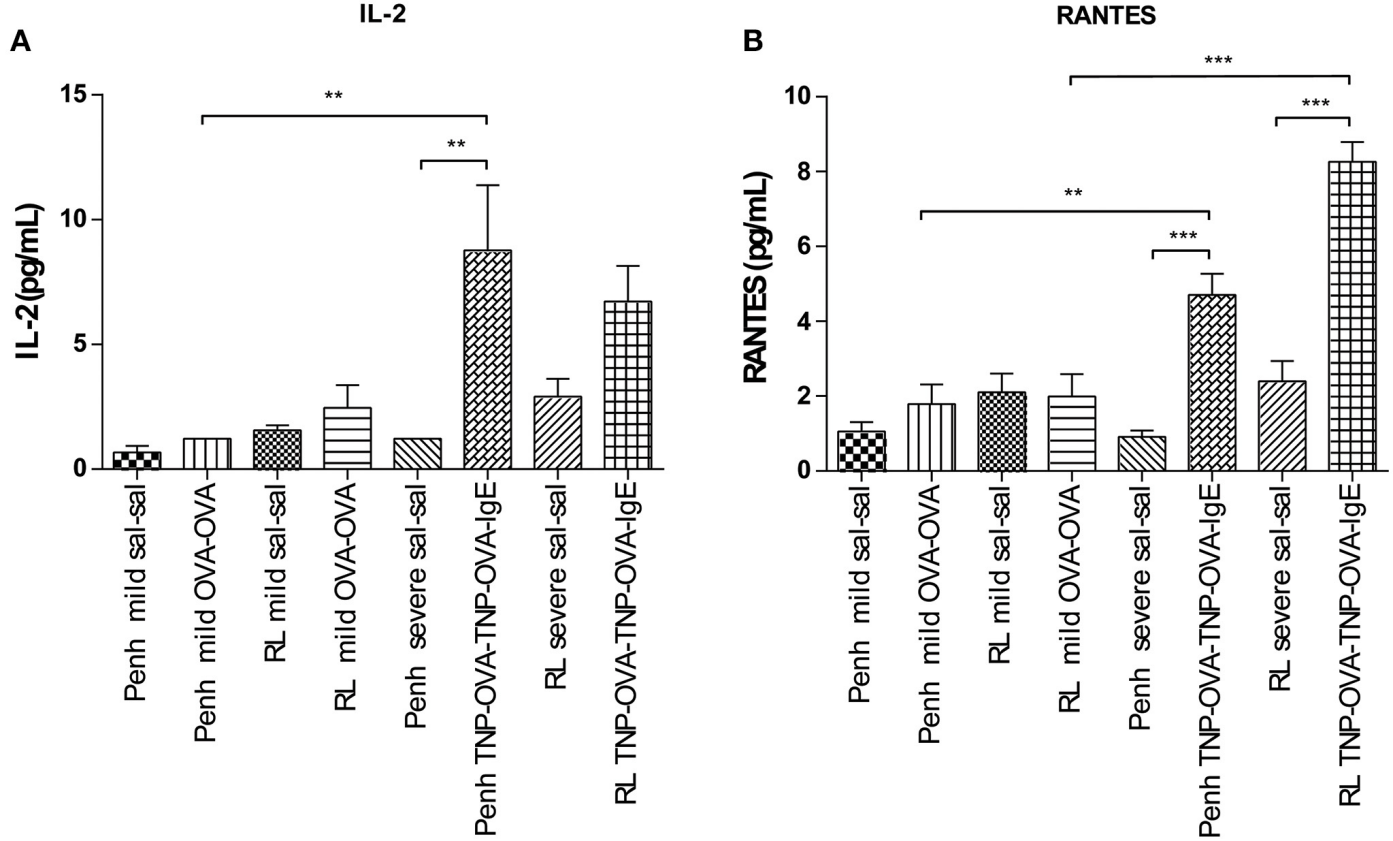
**Cytokine measurement in BALF**. Cytokines were measured in mice sensitized with saline or ovalbumin and challenged by aerosol with saline or ovalbumin (mild) and mice sensitized with saline or TNP-OVA and challenged intranasally with saline or TNP-OVA-IgE (severe) after Penh measurement or R_L_ measurement. **(A)** IL-2 concentration in pg/mL, **(B)** RANTES concentration in pg/mL. Values are expressed as mean ± s.e.m. ^**^P < 0.01, ^***^P < 0.001 using a One-Way ANOVA followed by a Bonferroni test, *n* = 4–5 mice/group.

Inflammatory cytokines were measured in the BALF of the experimental groups. There was no relation between the cytokine profile or amount of cytokine protein and ventilation vs. no ventilation. Therefore, in contrast to the number of inflammatory cells, the ventilation procedure did not influence the type and amount of cytokines produced. However, the levels of IL-2 and RANTES were increased in BALF in the severe airway inflammation model compared to the mild model (Figures 6A,B, respectively). In the both models GM-CSF, IFNγ, IL-12p70, IL-13, IL-17, and MIP-2 were below detection limit. All other cytokines and chemokines are depicted in Tables 5, 6.

**Table 5:** **Chemokine and cytokine concentration in broncho-alveolar lavage fluid in the mild airway inflammation model**.

	**Penh**				**R_L_**			
	**SAL-SAL**	**SAL-OVA**	**OVA-SAL**	**OVA-OVA**	**SAL-SAL**	**SAL-OVA**	**OVA-SAL**	**OVA-OVA**
IL-10	n.d.	n.d.	17.8 ± 10.6	n.d.	n.d.	13.13 ± 6.53	n.d.	n.d.
IL-12p40	n.d.	n.d.	n.d.	n.d.	n.d.	n.d.	n.d.	n.d.
IL-1b	n.d.	n.d.	n.d.	n.d.	n.d.	n.d.	n.d.	n.d.
IL-2	0.67 ± 0.26	1.21 ± 0.08	n.d.	n.d.	1.55 ± 0.20	1.45 ± 0.28	0.91 ± 0.27	2.46 ± 0.91
IL-4	n.d.	n.d.	n.d.	130.30 ± 75.96	n.d.	n.d.	7.06 ± 3.62	48.98 ± 12.80
IL-5	n.d.	n.d.	n.d.	133.50 ± 53.23	n.d.	n.d.	35.85 ± 21.21	107.60 ± 71.93
IL-6	6.13 ± 4.91	n.d.	n.d.	3.90 ± 2.68	n.d.	n.d.	n.d.	n.d.
IL-9	49.04 ± 32.71	94.25 ± 61.92	n.d.	n.d.	n.d.	n.d.	41.45 ± 22.24	n.d.
IP-10	n.d.	n.d.	n.d.	5.92 ± 0.05	n.d.	n.d.	n.d.	74.58 ± 16.05
KC	37.92 ± 9.03	22.16 ± 4.85	29.06 ± 7.80	143.90 ± 40.57	35.43 ± 3.81	61.52 ± 9.12	74.17 ± 13.99	267.9 ± 92.75
MCP-1	n.d.	n.d.	n.d.	n.d.	n.d.	n.d.	n.d.	35.51 ± 16.98
MIG	n.d.	5.35 ± 1.71	n.d.	3.80 ± 1.59	n.d.	5.40 ± 1.80	2.02 ± 0.61	118.90 ± 41.71
MIP-1α	n.d.	n.d.	n.d.	83.37 ± 52.98	n.d.	n.d.	n.d.	132.80 ± 3.26
MIP-2	n.d.	n.d.	n.d.	n.d.	n.d.	n.d.	n.d.	n.d.
RANTES	1.06 ± 0.25	1.38 ± 0.39	1.03 ± 0.23	1.78 ± 0.52	2.11 ± 0.50	2.18 ± 0.22	1.67 ± 0.34	1.99 ± 0.60
TNFα	1.57 ± 0.33	2.06 ± 0.42	1.98 ± 0.03	1.65 ± 0.19	1.72 ± 0.23	2.21 ± 0.25	1.69 ± 0.10	1.66 ± 0.29

**Table 6:** **Chemokine and cytokine concentration in broncho-alveolar lavage fluid in the severe airway inflammation model**.

	**Penh**				**R_L_**			
	**SAL-SAL**	**SAL—TNP-OVA-IgE**	**TNP-OVA—SAL**	**TNP-OVA—TNP-OVA-IgE**	**SAL-SAL**	**SAL—TNP-OVA-IgE**	**TNP-OVA—SAL**	**TNP-OVA—TNP-OVA-IgE**
IL-10	n.d.	n.d.	n.d.	n.d.	7.83 ± 3.06	n.d.	16.23 ± 9.86	6.12 ± 4.90
IL-12p40	n.d.	n.d.	n.d.	n.d.	4.93 ± 2.19	n.d.	n.d.	33.87 ± 10.30
IL-1b	n.d.	n.d.	n.d.	n.d.	5.98 ± 2.94	n.d.	n.d.	9.56 ± 5.65
IL-2	n.d.	n.d.	n.d.	7.26 ± 2.52	2.91 ± 0.71	1.28 ± 0.35	2.52 ± 0.94	6.72 ± 1.42
IL-4	n.d.	n.d.	n.d.	5.51 ± 1.86	n.d.	n.d.	n.d.	5.14 ± 2.06
IL-5	n.d.	n.d.	n.d.	28.67 ± 14.7	n.d.	n.d.	n.d.	41.69 ± 26.33
IL-6	n.d.	n.d.	n.d.	n.d.	n.d.	n.d.	n.d.	n.d.
IL-9	n.d.	n.d.	n.d.	n.d.	n.d.	n.d.	n.d.	n.d.
IP-10	n.d.	n.d.	n.d.	42.00 ± 6.08	n.d.	101.40 ± 25.48	n.d.	11.6 ± 5.61
KC	85.40 ± 22.11	28.24 ± 6.70	39.90 ± 2.66	169.30 ± 58.66	33.1 ± 8.04	38.36 ± 7.59	14.55 ± 1.19	92.89 ± 29.16
MCP-1	n.d.	n.d.	n.d.	n.d.	n.d.	n.d.	n.d.	28.29 ± 18.00
MIG	n.d.	n.d.	n.d.	30.40 ± 4.71	5.48 ± 1.82	289.20 ± 84.17	1.59 ± 0.26	11.43 ± 6.10
MIP-1α	n.d.	n.d.	n.d.	79.70 ± 38.74	27.62 ± 17.25	n.d.	n.d.	n.d.
RANTES	0.91 ± 0.17	1.68 ± 0.35	0.91 ± 0.21	4.70 ± 0.57	2.40 ± 0.54	0.99 ± 0.36	2.73 ± 0.55	8.26 ± 0.52
TNFα	2.23 ± 0.18	2.41 ± 0.56	2.12 ± 0.01	1.62 ± 0.16	2.40 ± 0.34	1.49 ± 0.23	1.74 ± 0.26	2.10 ± 0.71

## Discussion

**Figure 7:**
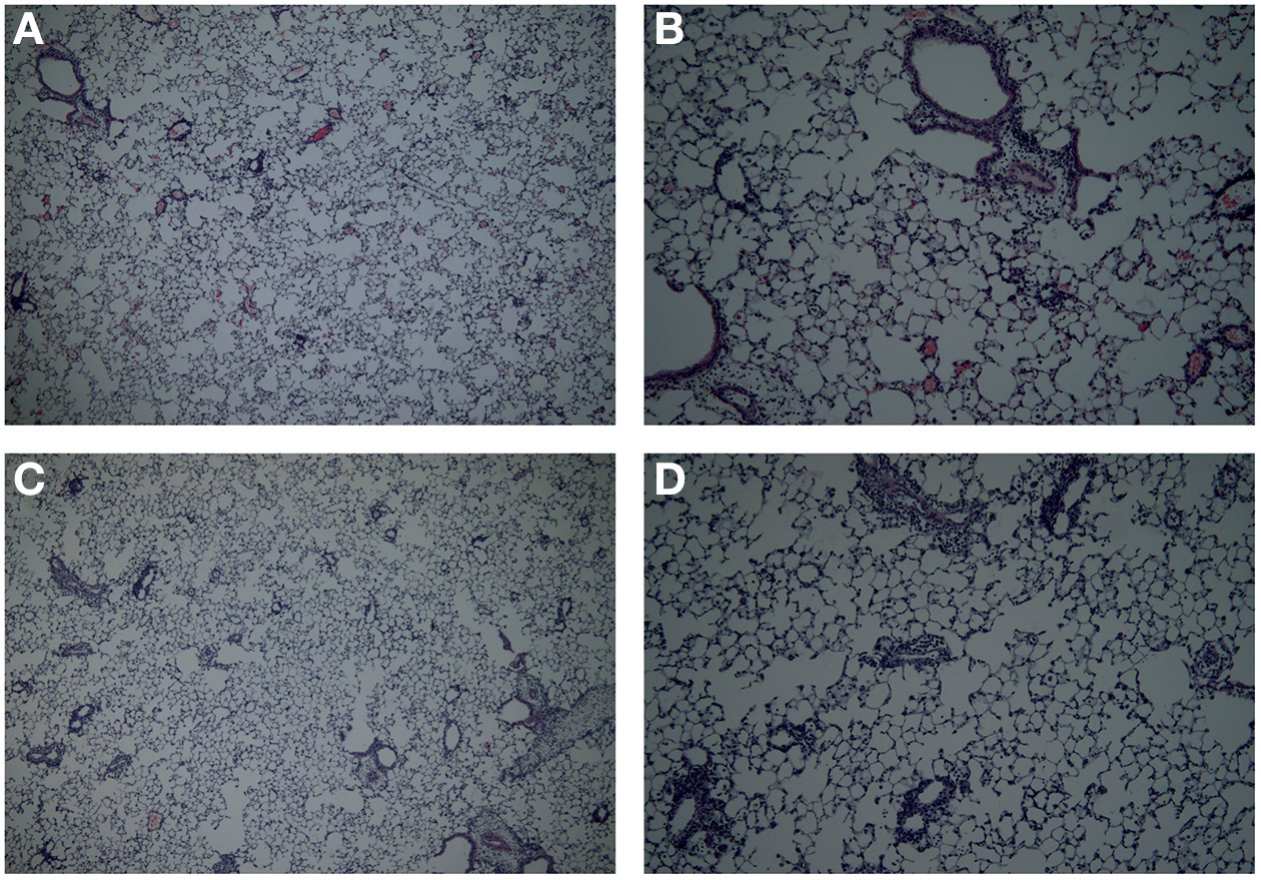
**Lung histology in the mild airway inflammation model**. Lungs were stained with H and E for histomorphometric analysis of the inflammation in mice sensitized with ovalbumin and challenged by aerosol with ovalbumin (mild) after Penh **(A,B)** or resistance measurement **(C,D)**. Magnification 40× **(A–C)** or 100× **(B–D)**.

In this study a comparison was made between the body plethysmography (Penh) and resistance (R_L_) measurements, to analyse murine airway function in two models for allergic airway inflammation. Although often referred to as a model for asthma these animal models do not reflect all pathophysiological mechanisms in asthma patients (Kumar and Foster, 2012). Therefor the models used in this manuscript are referred to as models of allergic airway inflammation. We demonstrated that the Penh measures are not as pronounced in the mild model as compared to R_L_ measures. However, an additional increase in inflammatory cells was found during the R_L_ measurement, which was most likely due to enforced pulmonary ventilation. In the severe inflammation model the increase in Penh was more pronounced compared to the R_L_. Using the bodyplethysmographic analysis of Penh, the total number of BAL cells in the OVA-OVA group increased three times compared to the SAL-SAL group in the mild inflammation model. The increase was mainly due to an increase in eosinophils. Eosinophilic inflammation were characteristic for allergic asthma (Barnes, 2008). In severe asthma, the numbers of eosinophils, macrophages, lymphocytes, and neutrophils are higher compared to mild asthma (Barnes, 2008). In agreement, in the severe inflammation model, the total number of BAL cells after TNP-OVA sensitization and TNP-OVA-IgE challenge was more than five times higher compared to OVA-OVA group in the mild inflammation model. The number of eosinophils in the severe airway inflammation model was more than seven times higher than in the mild inflammation model, as demonstrated earlier by Zuberi et al. (2000) and Sagar et al. (2013). After ventilation of the animals and R_L_ measurements, the total number of BAL cells was higher compared to Penh, both in the mild (OVA-OVA group) and severe inflammation model (TNP-OVA—TNP-OVA-IgE group). In the severe airway inflammation model this increase was not as high as in the mild inflammation model. Potentially due to the number of BAL cells already at a maximum level caused by the process: ventilator-induced lung injury (VILI). Cannizzaro et al. (2011) and Zhang et al. (2009) demonstrated that mechanical ventilation increases the total number of BAL cells in BALB/c mice. In the mild inflammation model, only a slight but not significant increase in the Penh in the OVA-OVA group was found. Resistance of the upper airways may influence the outcome of changes in the lower airways (Hoymann, 2006), explaining this anomaly. In contrast, a pronounced airway hyperresponsiveness was observed in the severe inflammation model. This indicates that Penh measurements might only be useful to investigate airway function under conditions of severe inflammation. When the airway responsiveness was measured using the R_L_ method, a significant increase in both the mild and severe allergic airway inflammation model was recorded. Strikingly, ventilation causes a significant increase in the number of BAL cells in the mild model, but not in the severe model. The number of inflammatory cells does not correlated with the airway hyperresponsiveness. From studies it is known that airway hyperresponsiveness to bronchoconstrictor agents does not correlate with inflammation, but indirect stimuli, like hypertonic saline does (Kumar and Foster, 2012). Histology of the lungs showed that more cells are present in mice with mild allergic airway inflammation after the R_L_ measurement as compared to these after Penh measurement (Figure 7). The increase in the number of BAL cells in the ventilated animals of the mild inflammation model could not be explained by changes in chemokines or cytokines, but significantly higher levels of IL-2 and RANTES were observed in the severe model compared to the mild model. IL-2 can be produced by epithelial cells and eosinophils (Chung and Barnes, 1999) and increased levels are found in the BALF of patients with symptomatic asthma (Chung and Barnes, 1999; Bloemen et al., 2007). These experiments are in line with our observation that more eosinophils were present in the severe model. The eosinophilia might be further promoted by RANTES. RANTES is a CC chemokine involved in the chemoattraction of T lymphocytes, monocytes and eosinophils (Chung and Barnes, 1999; Saad-El-Din Bessa et al., 2012). Increased levels of RANTES are present in the BALF obtained from asthmatic patients (Lukacs et al., 1997; Chung and Barnes, 1999; Saad-El-Din Bessa et al., 2012) and blocking antibodies against RANTES are able to inhibit airway inflammation in a murine model of allergic airway disease (Saad-El-Din Bessa et al., 2012). In conclusion, in models with mild inflammation, body plethysmography for the determination of the airway hyperresponsiveness may not be as reliable as measurements of resistance which provided a more accurate analysis compared with previous studies. In severe models with more pronounced airway inflammation, both body plethysmography and measurement of R_L_ may be used to analyze airway function. Along with the invasive procedure, a disadvantage of the R_L_ method could be the ventilation-induced increase in BAL cell numbers under mild inflammatory conditions.

## Funding

This project is jointly financed by the European Union, European Regional Development Fund and The Ministry of Economic Affairs, Agriculture and Innovation, Peaks in the Delta, the Municipality of Groningen, the Provinces of Groningen, Fryslân and Drenthe, the Dutch Carbohydrate Competence Center (CCC WP25;www.cccresearch.nl), Nutricia Research and FrieslandCampina.

### Conflict of interest statement

The authors declare that the research was conducted in the absence of any commercial or financial relationships that could be construed as a potential conflict of interest.

## Abbreviations

BALF: Broncho-Alveolar Lavage Fluid
OVA: Ovalbumin
R_L_: Lung Resistance
TNP: Trinitrophenyl
VILI: Ventilator-Induced Lung Injury.
